# Lipocalin-2-mediated astrocyte pyroptosis promotes neuroinflammatory injury via NLRP3 inflammasome activation in cerebral ischemia/reperfusion injury

**DOI:** 10.1186/s12974-023-02819-5

**Published:** 2023-06-23

**Authors:** Juanji Li, Pengfei Xu, Ye Hong, Yi Xie, Mengna Peng, Rui Sun, Hongquan Guo, Xiaohao Zhang, Wusheng Zhu, Junjun Wang, Xinfeng Liu

**Affiliations:** 1 Department of Neurology, Nanjing Jinling Hospital, Affiliated Hospital of Medical School, Nanjing University, Nanjing, 210002 Jiangsu China; 2 Division of Life Sciences and Medicine, Department of Neurology, The First Affiliated Hospital of USTC, University of Science and Technology of China, Hefei, 230001 Anhui China; 3 Department of Neurology, Nanjing First Hospital, Nanjing Medical University, Nanjing, 210002 Jiangsu China; 4 Department of Neurology, Shanghai Changhai Hospital, Second Military Medical University/Naval Medical University, Shanghai, 200433 China; 5 Department of Clinical Laboratory, Affiliated Jinling Hospital, Medical School of Nanjing University, 305# East Zhongshan Road, Nanjing, 210002 Jiangsu China

**Keywords:** Cerebral ischemia/reperfusion injury, Astrocyte, Pyroptosis, LCN2, NLRP3 inflammasome

## Abstract

**Background:**

Neuroinflammation is a vital pathophysiological process during ischemic stroke. Activated astrocytes play a major role in inflammation. Lipocalin-2 (LCN2), secreted by activated astrocytes, promotes neuroinflammation. Pyroptosis is a pro-inflammatory form of programmed cell death that has emerged as a new area of research in stroke. Nevertheless, the potential role of LCN2 in astrocyte pyroptosis remains unclear.

**Methods:**

An ischemic stroke model was established by middle cerebral artery occlusion (MCAO) in vivo. In this study, in vitro, oxygen–glucose deprivation and reoxygenation (O/R) were applied to cultured astrocytes. 24p3R (the LCN2 receptor) was inhibited by astrocyte-specific adeno-associated virus (AAV-GFAP-24p3Ri). MCC950 and Nigericin sodium salt (Nig) were used to inhibit or promote the activation of NLRP3 inflammasome pharmacologically, respectively. Histological and biochemical analyses were performed to assess astrocyte and neuron death. Additionally, the neurological deficits of mice were evaluated.

**Results:**

LCN2 expression was significantly induced in astrocytes 24 h after stroke onset in the mouse MCAO model. *Lcn2* knockout (*Lcn2*^−/−^) mice exhibited reduced infarct volume and improved neurological and cognitive functions after MCAO. LCN2 and its receptor 24p3R were colocalized in astrocytes. Mechanistically, suppression of 24p3R by AAV-GFAP-24p3Ri alleviated pyroptosis-related pore formation and the secretion of pro-inflammatory cytokines via LCN2, which was then reversed by Nig-induced NLRP3 inflammasome activation. Astrocyte pyroptosis was exacerbated in *Lcn2*^−/−^ mice by intracerebroventricular administration of recombinant LCN2 (rLCN2), while this aggravation was restricted by blocking 24p3R or inhibiting NLRP3 inflammasome activation with MCC950.

**Conclusion:**

LCN2/24p3R mediates astrocyte pyroptosis via NLRP3 inflammasome activation following cerebral ischemia/reperfusion injury.

**Supplementary Information:**

The online version contains supplementary material available at 10.1186/s12974-023-02819-5.

## Introduction

Cerebral ischemia/reperfusion injury triggers a significant inflammatory response [1]. Astrocytes have emerged as key players in neuroinflammation [2]. Previous studies have shown that astrocytes play a central role in inflammatory pathology by communicating with CNS-resident cells such as microglia [3–5] or CNS-infiltrating cells [6, 7]. In recent years, a new pro-inflammatory programmed cell death, pyroptosis, has emerged. It is a caspase-1-mediated programmed cell death, characterized by GSDMD membrane pore formation, cell swelling, and an efflux of cytoplasmic pro-inflammatory cytokines [8, 9]. Inflammasome activation, such as NLRP3 upregulation, is essential for pyroptosis [8]. To date, several studies have shown that astrocyte pyroptosis is induced by cerebral ischemia insult, and pyroptosis suppression exerts anti-inflammatory and neuroprotective effects [10–13]. However, the upstream regulator of astrocyte pyroptosis has not been reported to date.

Lipocalin-2 (LCN2), a member of the lipocalin family [14], functions as an acute-phase protein following brain injury [15, 16]. In the CNS, astrocytes are the primary source and target of LCN2 during brain injury [17, 18]. In a previous study, we showed that LCN2 induces astrocyte activation and exacerbates inflammatory injury in cerebral ischemia [19], which might function through pro-inflammatory cytokines [20, 21]. LCN2 also modulates cell death, including apoptosis [17, 22] and autophagy [23]. Nonetheless, the potential role of LCN2 in astrocyte pyroptosis needs to be further explored. Thus, the present study aimed to investigate whether LCN2 is involved in astrocyte pyroptosis and whether it is associated with NLRP3 inflammasome activation post-cerebral ischemia/reperfusion injury.

## Materials and methods

### Animals

Male C57BL/6J mice weighing 20–25 g were used for the in vivo experiments. Wild type (WT) mice were purchased from the Model Animal Research Institute of Nanjing University (Nanjing, China), and *Lcn2* knockout (*Lcn2*^−/−^) mice (B6.129P2-Lcn2tm1Aade/AkiJ, Jax) were provided by the Jackson Laboratory. All mice were housed under appropriate conditions (23 ± 2 °C, 55–60% relative humidity and a 12 h light/dark cycle) with free access to food and water.

### Animal model of cerebral ischemia/reperfusion injury

Transient middle cerebral artery occlusion (MCAO) was performed as described previously [24]. Briefly, mice were anesthetized with 2% isoflurane in O_2_. Then, the right common carotid artery (CCA), right external carotid artery (ECA) and right internal carotid artery (ICA) were separated through a middle anterior neck incision. CCA and ECA were ligated with a 6-0 suture. Next, we inserted a silicone-coated nylon thread (diameter of 0.16 ± 0.02 mm) from a small cut on the ECA into the ICA to block the bifurcation of the middle cerebral artery (MCA). After blocking the blood flow for 90 min, nylon thread was extracted for reperfusion. A heating pad was used to maintain body temperature during the whole procedure. For sham-operated mice, the same procedure was applied except for MCA occlusion.

### Brain infarct volume determination

The infarct volume was confirmed by 2,3,5-triphenyltetrazoliumchloride (TTC, Sigma, USA) staining 24 h after MCAO. The brain was sliced into 1-mm-thick sections and stained with 2% TTC solution at 37 °C for 15 min in the dark, followed by fixation with 4% paraformaldehyde (PFA) at 4 °C overnight [25]. Brain sections were scanned on a HP Scanjet G3110, and the relative infarct volume was assessed with the ImageJ software (1.50 g, Wayne Rasband, National Institutes of Health, USA). The relative infarct volume was derived by the following formula: (contralateral hemisphere volume − normal ipsilateral hemisphere volume)/(contralateral hemisphere volume × 2) × 100% [25].

### Evaluation of neurological deficits and behaviors

The modified neurological severity score (mNSS) was used to evaluate the neurological deficit after MCAO [26]. The mNSS assessment system consists of four types of tests, including motor, sensory, beam balance, and reflexes and abnormal movements. The mNSS score ranges from 0 to 18, with 0 and 18 indicating no neurological deficit and the most severe deficit, respectively.

The Morris water maze (MWM) was performed to evaluate spatial learning and memory ability [27]. A blindness test was conducted to exclude blind and severely dyskinetic mice on day 22 after MCAO. The four groups of mice (*n* = 10 per group) were trained to find the platform in four trials for 5 consecutive days at days 23–27. The trial was stopped when the mice found the platform within 60 s. In case of failure, the mice were guided to the platform and left on it for 10 s to increase their memory. The time to find the platform (escape latency) and the swimming path were recorded. On day 28, the platform was removed for a probe trial, and each trial lasted 60 s. The time spent in the target quadrant and platform crossings were tracked and analyzed with the ANY-maze video tracking software (Stoelting, USA).

### Adeno-associated virus (AAV) injection and drug administration

Astrocyte-specific AAV was constructed by Shanghai Genechem Co., Ltd (Shanghai, China) to interfere with 24p3R expression. The following sequences were used: AAV-GFAP-24p3Ri, 5′-TCTGTATCCTCAGCATCAT-3′; AAV-GFAP-GFP, 5′-AGATTCTCCGAACGTGTCACGT-3′. The titers of AAV-GFAP-24p3Ri and AAV-GFAP-GFP were 1.09 E+13 (copies/mL) and 1.56 E+13 (copies/mL), respectively. AAV-GFAP-24p3Ri or AAV-GFAP-GFP (5 µL) was injected into the right lateral ventricle (coordinates: *a*/*p*, + 0.8; *m*/*l*, + 1.2; *d*/*v*, − 2.0) 4 weeks before the MCAO surgery. Next, we injected 10 μL of 1 μg/mL recombinant LCN2 (rLCN2) (Abcam, UK) into the right lateral ventricle for 5 continuous days before the MCAO surgery [28]. Nigericin sodium salt (Nig) (Selleck, USA) and MCC950 (Selleck) were used as an inducer and an inhibitor of NLRP3 inflammasome activation, respectively. Mice were treated with Nig (4 mg/kg, i.p) [29] or MCC950 (50 mg/kg, i.p) [30] immediately following the MCAO surgery.

### Primary astrocytes culture

Primary cortical astrocytes were cultured and purified, as described previously [31]. Briefly, astrocytes were harvested from neonatal mice within 24 h after birth. The bilateral cerebral cortex was collected in Hank’s balanced salt solution (HBSS, Gibco, USA) on ice, and meninges and blood vessels were stripped under a microscope. The tissue was digested with 0.125% trypsin–EDTA (Gibco) at 37 °C for 15 min. The digestion was terminated with Dulbecco’s modified Eagle medium (DMEM, high-glucose, Gibco) containing 10% fetal bovine serum (FBS, Gibco). The sample was homogenized, filtered through a screen cloth (diameter, 100 μm), and clarified by centrifugation at 1000 rpm for 5 min. The resulting cell pellet was resuspended in complete medium (DMEM containing 10% FBS and 1% penicillin–streptomycin) and cultured in T75 cell culture flasks (Corning, USA) coated with poly-d-lysine (PDL, Sigma, USA) overnight. After 24 h, the medium was changed to remove non-adherent cells. Subsequently, half of the medium was changed every 3 days until complete cell fusion. In order to harvest the purified astrocytes, the cells were agitated at 37 °C and 400 rpm for 6 h. Floating cells were removed by washes with sterile phosphate-buffered saline (PBS). Adherent cells were digested with 0.25% trypsin–EDTA for 3 min at room temperature. The resuspended cells were seeded in cell culture dishes, and media were changed every 3 days.

### Oxygen–glucose deprivation/reoxygenation

For oxygen–glucose deprivation (OGD), the medium was replaced with glucose- and FBS-free DMEM (Gibco). The cells were incubated in an anaerobic chamber equipped with AnaeroPack-Anaero (MGC, Japan) at 37 °C. According to the time point reported previously [19], astrocytes were returned to normal culture conditions for reoxygenation after 6 h (OGD/R).

### Western blotting (WB)

Total protein was extracted from the penumbra region and cultured astrocytes with the RIPA lysis buffer (Cell Signaling Technology, USA) for WB, as reported previously [32]. Protein concentration was detected with the BCA kit (Generay Biotechnology, China). Total protein was separated by 8–12% sodium dodecyl sulfate-polyacrylamide gel electrophoresis (SDS-PAGE) and the protein bands were transferred onto polyvinylidene difluoride (PVDF) membranes (Millipore, USA). Then, the membranes were probed at 4 °C overnight with primary antibodies raised against GFAP (1:5000, Abcam), LCN2 (1:200, Abcam), 24p3R (1:500, Biorbyt, UK), NLRP3 (1:500, Abcam), ASC (1:500, Santa Cruz Biotechnology, USA), caspase-1 (1:500, Abcam), GSDMD (1:500, Santa Cruz Biotechnology), IL-1β (1:500, Santa Cruz Biotechnology), IL-18 (1:500, Abcam) and β-actin (1:5000, Cell Signaling Technology, USA). Subsequently, the membranes were incubated with the corresponding horseradish peroxidase (HRP)-conjugated secondary antibodies at room temperature for 1 h. The enhanced chemiluminescence kit (Millipore, USA) was used for detection, and the ImageJ software was used for quantitation. β-Actin served as a loading control.

### Immunofluorescence (IF) and TUNEL staining

The mice were killed after perfusion, and the brains were fixed with PFA at 4 °C overnight. Dehydration was carried out with a sucrose gradient (10%, 20%, and 30%) at 4 °C. After freezing in an optimal cutting temperature compound (Sakura Finetek, Japan), the brains were sliced into 10-μm sections for IF.

Brain slices and cell-climbing sheets were washed with PBS before fixation with PFA at room temperature for 10 min. Then, the slices were blocked with 1% bovine serum albumin (BSA; AMRESCO, USA) containing 0.1% Triton-100 (Sigma) and 10% goat serum (Beyotime, China) for 1 h. Then, the samples were incubated with primary antibody against GFAP (1:1000, Abcam), NeuN (1:500, Abcam), LCN2 (1:200, Abcam), 24p3R (1:100, Biorbyt, UK), NLRP3 (1:200, Abcam), ASC (1:200, Santa Cruz Biotechnology), caspase-1 (1:500, Abcam) and GSDMD (1:200, Santa Cruz Biotechnology) at 4 °C overnight, followed by incubation with appropriate Alexa Fluor-488/594/647-conjugated secondary antibodies (Jackson ImmunoResearch, USA) for 1 h in the dark. After PBS washes, the slices were mounted with CC/MOUNT (Sigma). Images were captured under a fluorescence microscope (Olympus MX51, Japan) or a FluoView FV3000 confocal laser scanning microscope (Olympus).

In order to detect neuron apoptosis, TdT-mediated dUTP nick-end labeling (TUNEL) was performed with One-Step TUNEL Assay Kit (Beyotime). Briefly, slices were incubated with anti-NeuN primary antibody (1:500, Abcam) at 4 °C overnight, followed by TUNEL staining in the dark at 37 °C for 1 h. DAPI (1:1000, Sigma) counterstaining was used to label the nuclei. The ratio of apoptotic neurons was calculated with the ImageJ software. The percentage of TUNEL-positive neurons (red) relative to total NeuN-stained neurons (green) was calculated to assess neuronal apoptosis. Four regions were selected for analysis in each sample, and the average ratio was computed.

### Enzyme-linked immunosorbent assay (ELISA)

LCN2, LDH, IL-1β and IL-18 were measured in astrocyte culture supernatants with specific ELISA kits (R&D system, USA; Abcam and NEOBIOSCIENCE, China) according to the manufacturer’s instructions. Absorbance was measured at 450 nm on a microplate reader (Thermo Fisher Scientific, USA).

### Real-time quantitative polymerase chain reaction (qPCR)

Total RNA was extracted with TRIzol reagent (Sigma) from a peri-infarct region of brains and cultured astrocytes, and reverse transcribed into cDNA with RevertAid First Strand cDNA Synthesis Kit (Thermo Scientific). A 25-μL reaction system consisted of diluent cDNA (1:10), specific primers and UItraSYBR Mixture (CWBio, China). Amplification was carried out on a Stratagene Mx3000P QPCR system (Agilent Technologies, USA). *GAPDH* served as an endogenous control. The primer pairs used in this study were: *24p3R*-forward 5′-TACCTGATGCGCCTGGAGCT-3′ and *24p3R*-reverse 5′-TTCTCCAGTTCCTGCAAAGCTT-3′; *GAPDH*-forward 5′-AAGAAGGTGGTGAAGCAGG-3′ and *GAPDH*-reverse 5′-GAAGGTGGAAGAGTGGGAGT-3′.

### Transmission electron microscopy (TEM)

Brain tissues were fixed with the electron microscopy fixative and processed as described previously [33]. The sections were scanned with an H7500 transmission electron microscope (Hitachi, Japan).

### Statistical analysis

All statistical analyses were performed with the SPSS software (vision 22.0, SPSS Inc., IBM, NY, USA). Continuous variables are mean ± standard deviation (SD). Two-way repeated-measures analysis of variance (ANOVA) followed by Tukey’s post hoc test was used to analyze escape latency and the swimming path in the MWM test. Other data were analyzed by one-way ANOVA followed by Tukey’s post hoc test. Statistical significance was considered at *P* < 0.05.

## Results

### LCN2 expression in astrocytes is induced by ischemia/reperfusion injury

**Fig. 1:**
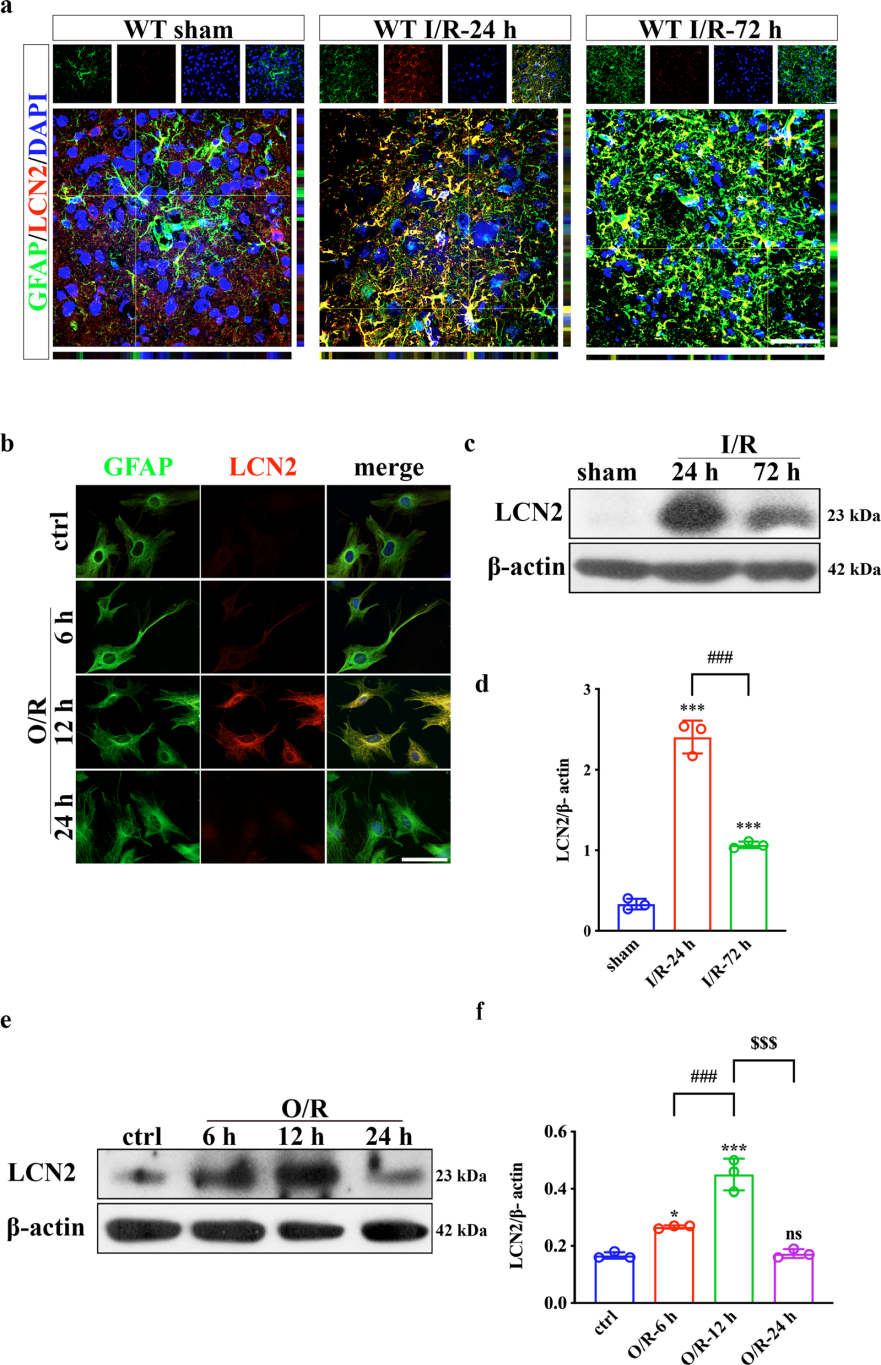
LCN2 in astrocytes is upregulated following ischemia/reperfusion injury both in vivo and in vitro. **a**, **b** Double-label IF staining of GFAP (green) and LCN2 (red) in the peri-infarct region and OGD/R-treated astrocytes. Scale bar = 20 μm. **c**–**f** Immunoblotting analysis of LCN2 expression after MCAO or OGD/R. Data are mean ± SD; *n* = 3 per group; ***P < 0.001, *P < 0.05 vs. sham-operated or control group; ^###^*P* < 0.001, ^$$$^*P* < 0.001

Reactive astrogliosis occurs after ischemic stroke through morphological changes and increased GFAP expression [34]. According to previous findings, reactive astrogliosis mostly occurs in the penumbra region [35, 36]. The current data were consistent with previous studies (Additional file 1: Fig. S1). At 24 h and 72 h post-MCAO surgery, IF and WB consistently revealed that GFAP expression was remarkably increased, indicating successful activation of astrocytes. Co-IF staining confirmed that LCN2 was upregulated by ischemia and colocalized with GFAP (Fig. 1a, b). Interestingly, LCN2 expression peaked at 24 h post-MCAO and decreased subsequently (Fig. 1c, d). In the in vitro model of ischemic stroke, the maximum level of astrocyte LCN2 expression appeared at 12 h after OGD/R (Fig. 1e, f). This phenomenon was consistent with ELISA findings reflecting LCN2 secretion into culture supernatants (Additional file 1: Fig. S2a). LDH release assay found no significant difference in cell death after reoxygenation for different periods. The effect of cell activity on LCN2 release could be ruled out (Additional file 1: Fig. S2b). Therefore, we selected 24 h in vivo and 12 h in vitro as the optimal time points for subsequent experiments.

### *Lcn2* knockout decreases brain damage and alleviates post-stroke neurological deficits

**Fig. 2:**
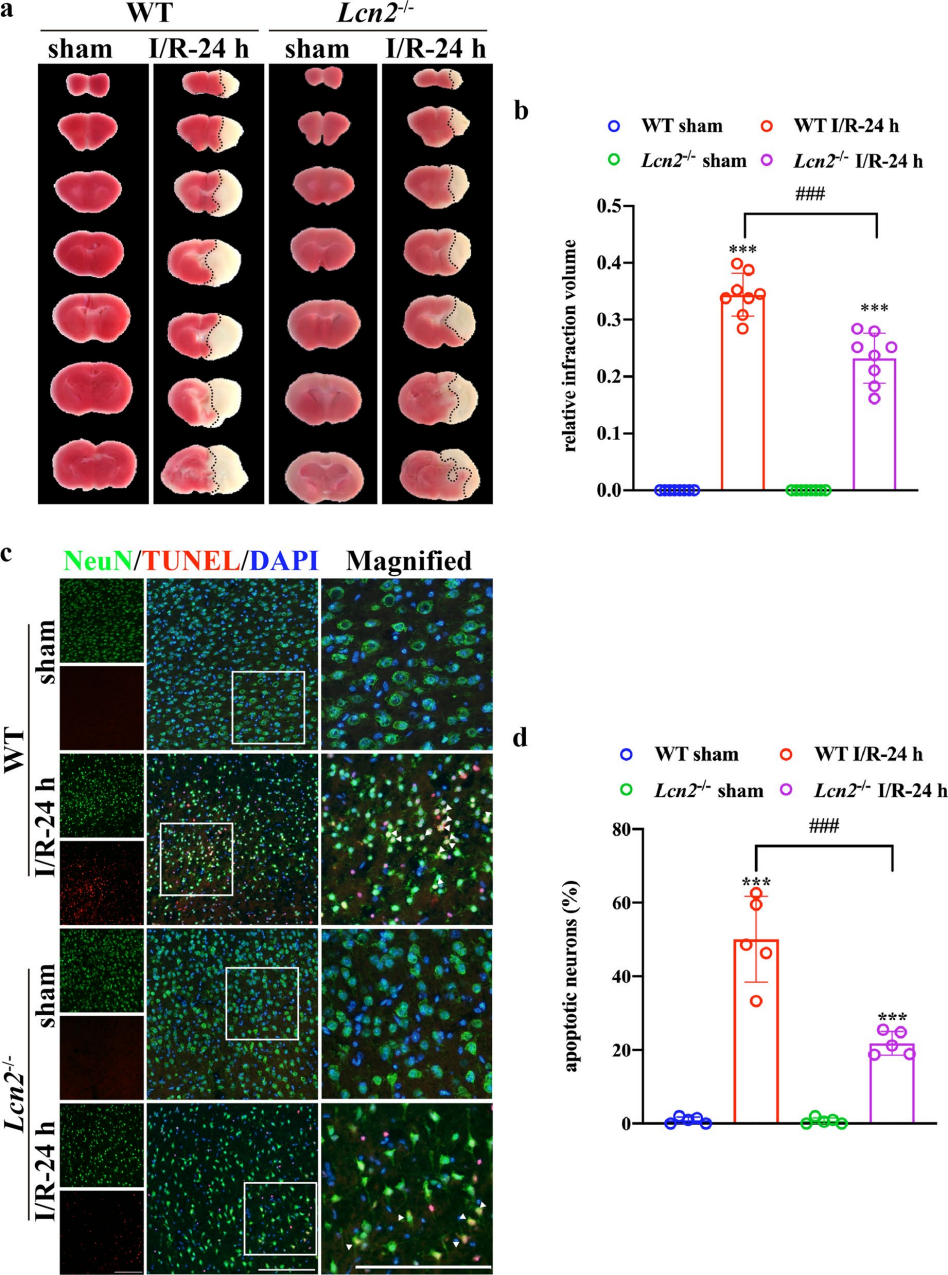
*Lcn2* knockout reduces infarct volume and neuronal death after MCAO surgery. **a**, **b** Infarct volume was assessed by TTC (2,3,5-triphenyltetrazoliumchloride) staining 24 h after the MCAO surgery (*n* = 10 per group). **c**, **d** Representative images of TUNEL double-staining (red) with NeuN (green) to assess the proportion of apoptotic neurons after the MCAO surgery. Scale bar = 50 μm. Data are mean ± SD; ***P < 0.001 vs. corresponding sham-operated group; ^###^*P* < 0.001

**Fig. 3:**
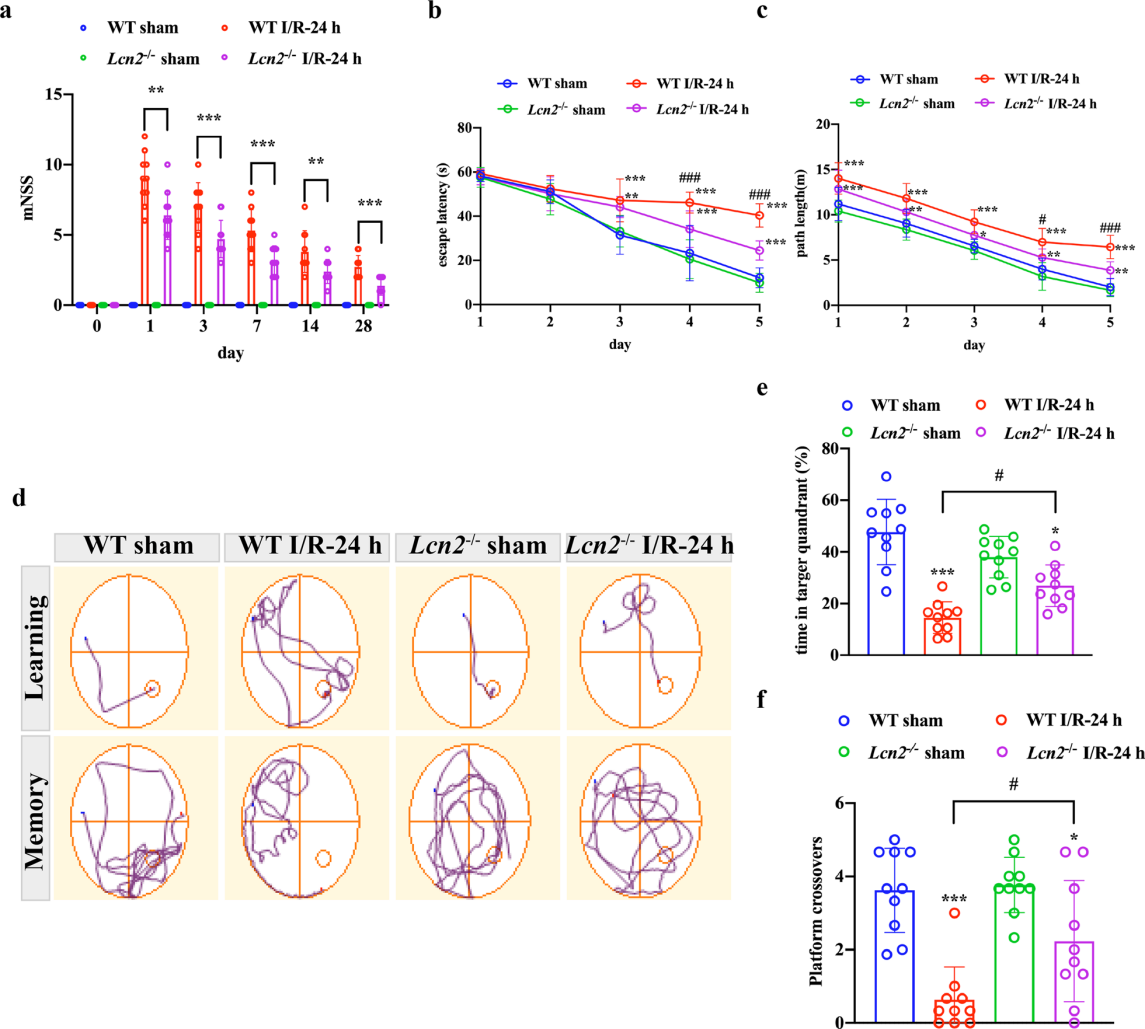
*Lcn2* deletion alleviates post-stroke neurological deficits. **a** mNSS was used for sensorimotor function assessment after the MCAO surgery. ***P < 0.001, **P < 0.01 for MCAO-operated mice in WT group vs. *Lcn2*^−/−^ group. **b**–**f** Long-term cognitive function was assessed by the MWM at days 22–28 post-MCAO surgery. **d** Representative swimming paths of mice attempting to find the hidden platform on the last training day (top traces, “learning”) or searching for the removed platform in the probe trial (bottom traces, “memory”). **b**, **c** Escape latency and path length were recorded on days 23–27 post-MCAO surgery. ***P < 0.001, **P < 0.01, *P < 0.05 for MCAO-operated group vs. corresponding sham-operated group; ^###^*P* < 0.001, ^#^*P* < 0.05 for MCAO-operated mice in WT group vs. *Lcn2*^−/−^ group. **e**, **f** Time in the target quadrant and platform crossings were recorded in the probe trial, ***P < 0.001 and *P < 0.05 for MCAO-operated group vs. corresponding sham-operated group; ^#^*P* < 0.05. Data are mean ± SD; *n* = 10 per group

We applied TTC staining to evaluate infarct volume 24 h after MCAO. The results demonstrated that after *Lcn2* gene knockout, infarct volume decreased dramatically from 34.40 ± 3.76% to 23.24 ± 4.42% (Fig. 2a, b; *P* < 0.001). Next, we investigated whether *Lcn2* deletion affects ischemia/reperfusion-induced neuronal apoptosis. Hence, NeuN/TUNEL co-immunostaining was used to detect neuronal apoptosis in the penumbra (Fig. 2c). About 50.06 ± 11.66% TUNEL-positive neurons were detected in the peri-infarct region in WT mice, while *Lcn2* deletion significantly reduced TUNEL-positive neurons in the penumbra to 21.82 ± 3.20% (Fig. 2d, *P* < 0.001). mNSS was used to assess post-stroke neurological function on post-stroke days 1, 3, 7, 14 and 28. As shown in Fig. 3a, mice exposed to MCAO recovered gradually but with permanent sensorimotor deficits. However, *Lcn2* gene deficiency significantly improved post-stroke sensorimotor function compared with the WT group (*P* < 0.001 for days 1, 3 and 7; *P* = 0.007 for day 14; *P* = 0.014 for day 28). In addition, we used the MWM to assess spatial learning and memory function at post-surgery days 22–28. The prolonged training time decreased escape latency and path length to the platform in all groups. Nevertheless, *Lcn2*^−/−^ mice performed better than WT mice. In the training trial, *Lcn2* deficiency reduced escape latency and shortened path length compared with WT MCAO mice (Fig. 3b, c, *P* < 0.001 for both on the last training day). The probe trial proceeded the day after the last training day. *Lcn2*^−/−^ mice crossed the hidden platform more times and spent more time in the target quadrant compared with WT MCAO mice (Fig. 3d–f, *P* < 0.05 for both). Strikingly, no significant differences were observed between the sham-operated groups for WT or *Lcn2*^−/−^ animals.

### *Lcn2* deficiency alleviates pyroptosis in astrocytes induced by ischemia/reperfusion injury

**Fig. 4:**
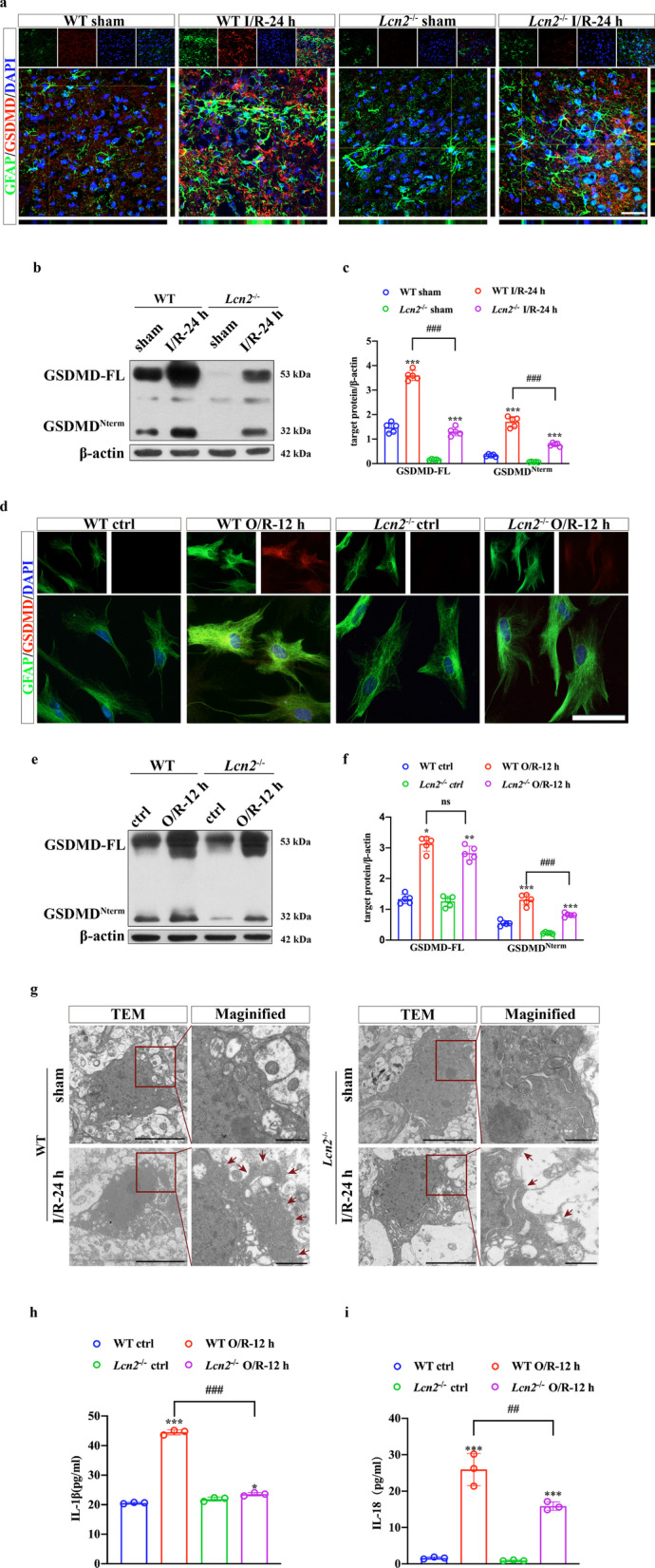
*Lcn2* deficiency alleviates pyroptosis in astrocytes induced by ischemia/reperfusion injury. **a**, **d** Double-IF staining for GFAP (green) and GSDMD (red) in vivo and in vitro. Scale bar = 20 μm. **b**, **c**, **e**, **f** WB analysis of GSDMD protein expression in the penumbra tissue and cultured astrocytes. Data are mean ± SD; *n* = 5 per group; ***P < 0.001, **P < 0.01, *P < 0.05 for MCAO-operated group vs. corresponding sham-operated group or OGD/R-cells vs. corresponding control cells; ^###^*P* < 0.001. **g** Representative TEM images of astrocytes in peri-infarct brain tissue. Red arrows indicate pyroptosis pores on the cell membrane. Scale bar = 5 μm and 1 μm for magnified images. **h**, **i** IL-1β and IL-18 secretion into supernatants measured by ELISA. Data are mean ± SD; *n* = 3 per group; ***P < 0.001 for OGD/R-cells vs. corresponding control cells; ^###^*P* < 0.001, ^##^*P* < 0.01

Since GSDMD is a marker of pyroptosis expressed on the membrane, we detected the expression of GSDMD by IF and WB in vivo and in vitro. As shown in Fig. 4a, GSDMD was scarcely expressed in the sham-operated group, but the expression of GSDMD was elevated by the MCAO operation. However, GSDMD expression was reduced by *Lcn2* knockout. In vitro findings revealed that GSDMD was expressed on the membrane of astrocytes after OGD exposure. *Lcn2*^−/−^ astrocytes showed reduced GSDMD expression compared with WT astrocytes (Fig. 4d). The expression of the pore-forming protein GSDMD^Nterm^ was increased after ischemia/reperfusion injury, which could be suppressed by *Lcn2* knockout (Fig. 4b, c, e, f, *P* < 0.001 for both in vivo and in vitro). These results demonstrated that *Lcn2* deficiency alleviates pyroptosis in astrocytes.

The pyroptosis pores on astrocytes were examined by electron microscopy. GSDMD^Nterm^-formed pores were clearly visible on the membrane of astrocytes with ischemia/reperfusion injury. Membrane pores were less frequent in the *Lcn2*^−/−^ group compared with the WT group (Fig. 4g). As IL-1β and IL-18 could be released through GSDMD^Nterm^ pores on the membrane of astrocytes [37], we collected cell culture supernatants to evaluate IL-1β and IL-18 secretion in astrocytes. ELISA suggested that the upregulated secretion of IL-1β and IL-18 was reversed by *Lcn2* knockout (Fig. 4h, i, *P* < 0.001 for IL-1β and *P* = 0.003 for IL-18).

### LCN2 induces NLRP3 inflammasome activation and astrocyte pyroptosis via binding to 24p3R

**Fig. 5:**
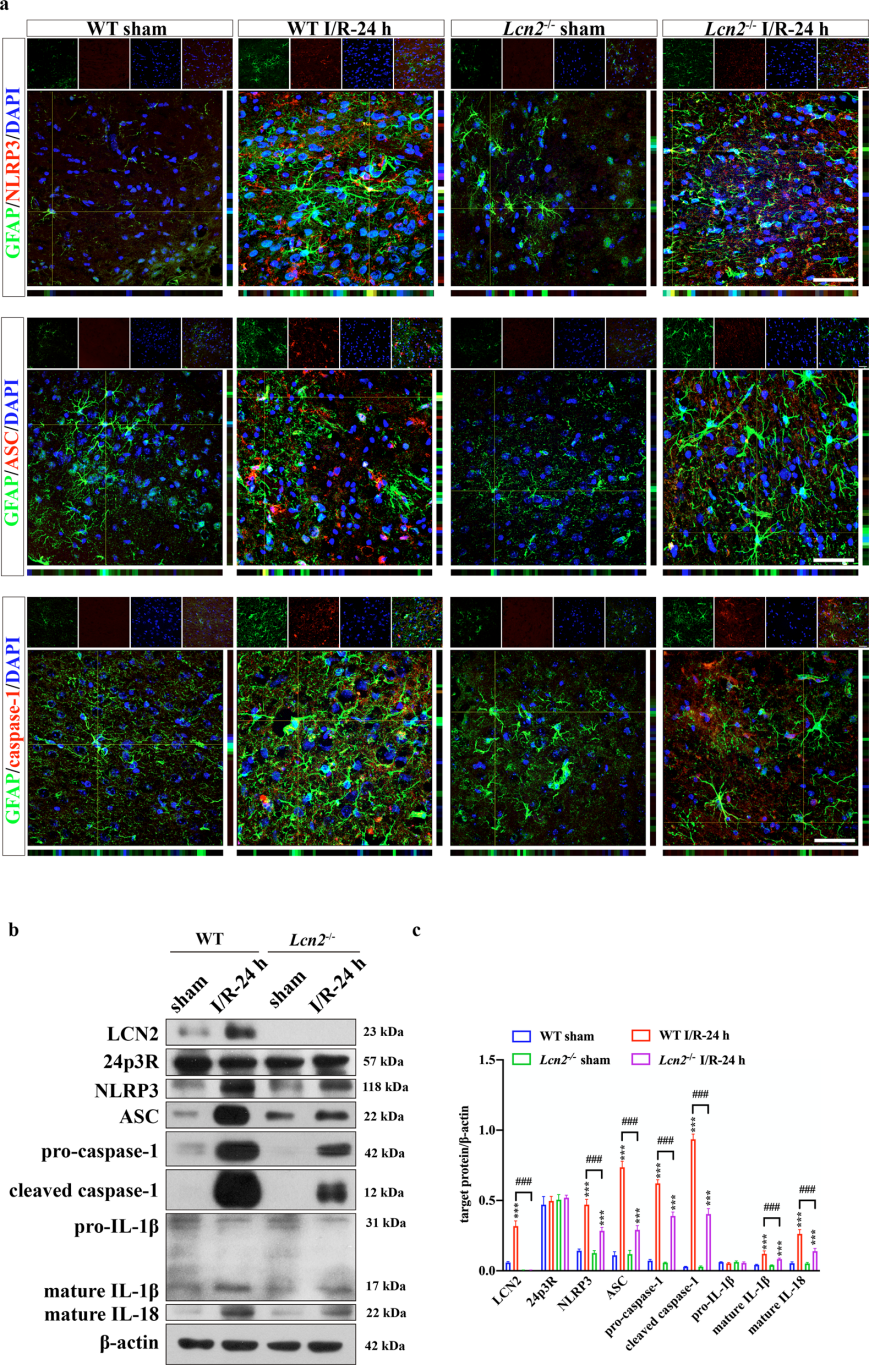
LCN2 induces NLRP3 inflammasome activation and subsequent pyroptosis in astrocytes. **a** Co-IF staining of GFAP with NLRP3, ASC and caspase-1. Scale bar = 20 μm. **b** WB analysis of LCN2, 24p3R, NLRP3, ASC, pro-caspase-1, cleaved caspase-1, pro-IL-1β, mature IL-1β and mature IL-18 in the penumbra region. Data are mean ± SD; *n* = 5 per group; ***P < 0.001 vs. corresponding sham-operated group; ^###^*P* < 0.001

GSDMD is cleaved into GSDMD^Nterm^ by activated NLRP3 inflammasome, which is essential for the formation of pyroptosis pores [38]. Then, we investigated whether LCN2 induces astrocyte pyroptosis through NLRP3 inflammasome activation by IF and WB. As shown in Fig. 5, the increased expression of the target proteins of the NLRP3 inflammasome activation pathway, including ASC, pro-caspase-1, cleaved caspase-1, mature IL-1β and mature IL-18, could be significantly suppressed by *Lcn2* knockout (*P* < 0.001 for all the above target proteins).

**Fig. 6:**
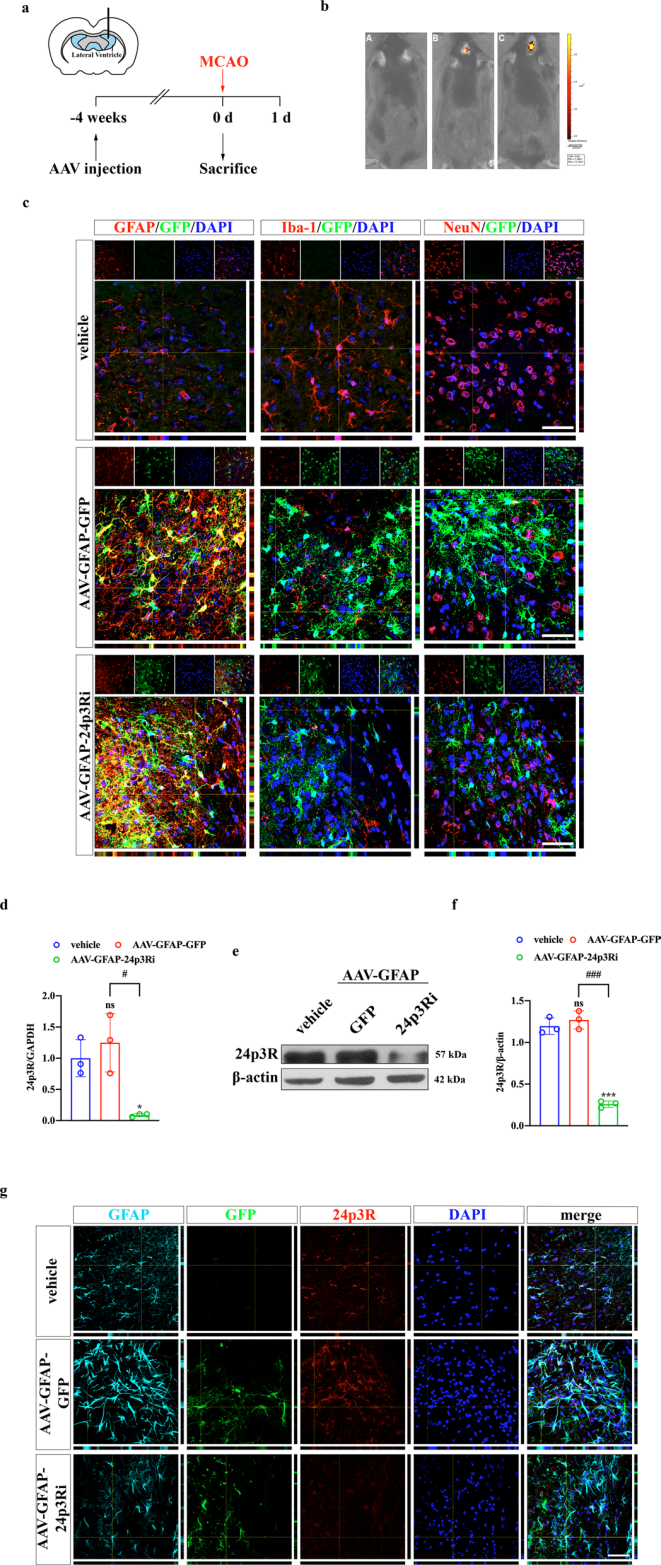
AAV-GFAP-24p3Ri transfection reduces 24p3R expression in astrocytes. **a** Schematic representation of the intracerebroventricular injection procedure. **b** Representative images of the small animal imaging system. **A** WT MCAO-operated mice. **B** WT MCAO-operated mice with AAV-GFAP-GFP injection. **C** WT MCAO-operated mice with AAV-GFAP-24p3Ri injection. **c** Representative images of AAV (labeled with GFP, green) specifically transfected into astrocytes but not microglia and neurons (labeled with GFAP, Iba-1 and NeuN, red). Scale bar = 20 μm. **d** qPCR was used to verify the efficacy of 24p3Ri at the transcriptional level. *n* = 3 per group; *P < 0.05 vs. vehicle-injection group; ^#^*P* < 0.5. **e**, **f** Detection of 24p3R expression after interference at the translational level by WB. n = 3 per group; ***P < 0.01 vs. vehicle-injection group; ^###^*P* < 0.001. **g** IF showed that astrocytic-24p3R expression was significantly inhibited by AAV-GFAP-24p3Ri injection. Scale bar = 20 μm

**Fig. 7:**
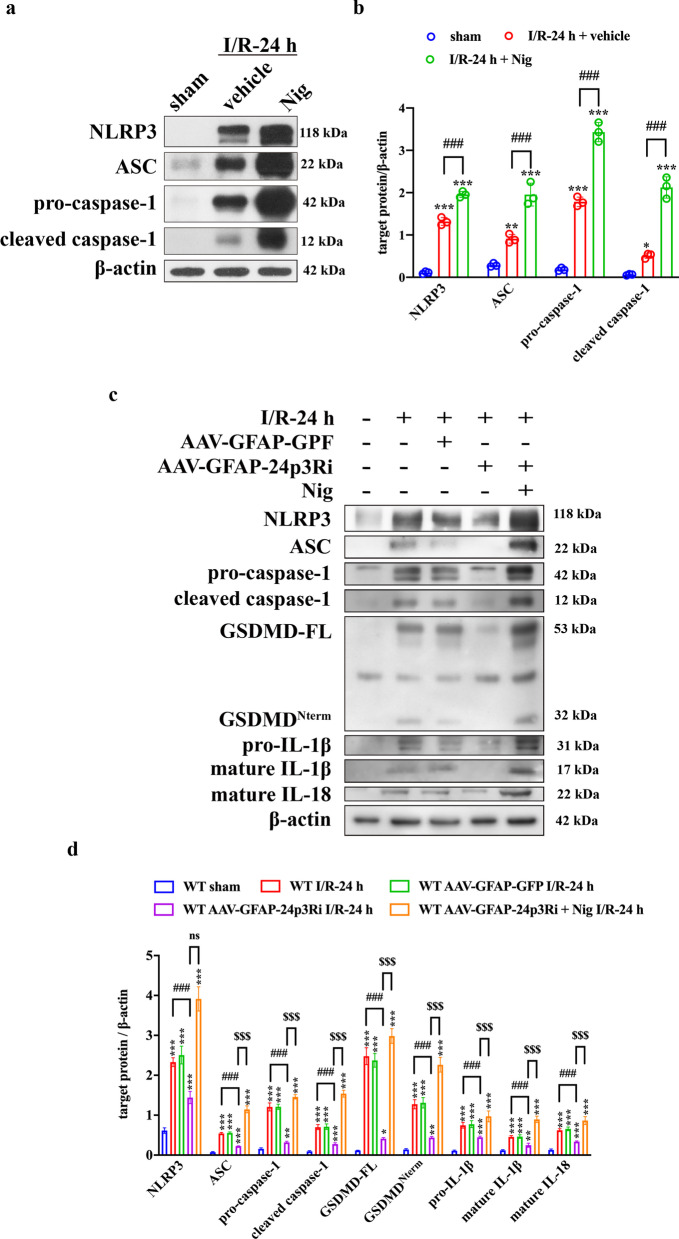
LCN2 mediates astrocyte pyroptosis by NLRP3 inflammasome activation via binding to 24p3R. **a**, **b** The efficacy of Nig in inducing NLRP3 inflammasome activation was evaluated by WB. *n* = 3 per group; ***P < 0.001, **P < 0.01; *P < 0.05 vs. sham-operated group; ^###^*P* < 0.001. **c**, **d** WB analysis of NLRP3, ASC, pro-caspase-1, cleaved caspase-1, GSDMD-FL, GSDMD^Nterm^, pro-IL-1β, mature IL-1β and mature IL-18. *n* = 5 per group; ***P < 0.001, **P < 0.01 vs. sham-operated group; ^###^*P* < 0.001; ^$$$^*P* < 0.001. Data are mean ± SD

Next, we investigated whether LCN2 function in the NLRP3 inflammasome was associated with its canonical receptor, 24p3R. IF confirmed that LCN2 and 24p3R were colocalized in astrocytes (Additional file 1: Fig. S3). We constructed astrocyte-specific AAV-24p3Ri (AAV-GFAP-24p3Ri) to interfere with 24p3R expression. AAV-GFAP-GFP and AAV-GFAP-24p3Ri (1.09 E+13 copies/mL, 1.56 E + 13 copies/mL, 2 μL) were administered by intracerebroventricular injection 4 weeks before the MCAO surgery (Fig. 6a). A small animal imaging system and immunostaining of brain sections were used to confirm that AAV was successfully injected into the lateral ventricle and transfected into astrocytes specifically (Fig. 6b, c). Intracerebroventricular injection of AAV-GFAP-24p3Ri significantly suppressed 24p3R expression both at the transcriptional and translational levels compared with AAV-GFAP-GFP injection (Fig. 6d–g). The NLRP3 inflammasome activation inducer, Nig (4 mg/kg), was injected intraperitoneally immediately after the MCAO surgery. NLRP3 inflammasome activation was enhanced by Nig administration (Fig. 7a, b). WB indicated that NLRP3 inflammasome activation and subsequent pyroptosis were inhibited by 24p3R interference. NLRP3 inflammasome activation by Nig could reverse 24p3R interference-induced suppression of astrocyte pyroptosis (Fig. 7c, d). Together, these findings suggested the association between LCN2 and 24p3R was essential in NLRP3 activation-induced astrocyte pyroptosis.

### NLRP3 inhibitor abolishes the effects of rLCN2 on astrocyte pyroptosis

**Fig. 8:**
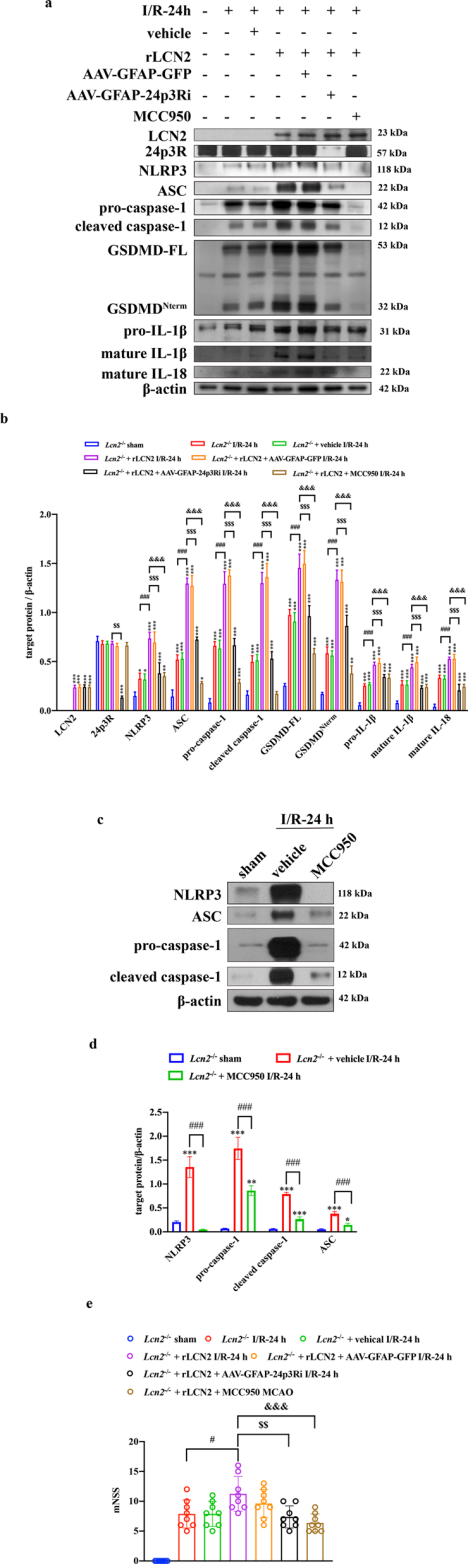
NLRP3 inhibitor abolishes the detrimental effects of rLCN2 on pyroptosis and functional prognosis. **a**, **b** WB analysis of LCN2, 24p3R and target protein in the NLRP3 inflammasome and pyroptosis pathway. *n* = 5 per group; ***P < 0.001, **P < 0.01, *P < 0.05 vs. sham-operated group; ^###^*P* < 0.001; ^$$$^*P* < 0.001, ^$$^*P* < 0.01; ^&&&^*P* < 0.001. **c**, **d** Detection of MCC950 function by WB analysis of NLRP3 expression. *n* = 3 per group; ***P < 0.001, **P < 0.01, *P < 0.05 vs. sham-operated group; ^###^*P* < 0.001. **e** Neurological impairment assessment 24 h after MCAO. *n* = 8 per group; ^#^*P* < 0.05; ^$$^*P* < 0.01; ^&&&^*P* < 0.001. Data are mean ± SD

rLCN2 was injected intraperitoneally (10 μL, 10 μg/mL) into *Lcn2*^−/−^ mice for 5 consecutive days before the MCAO surgery. rLCN2 administration aggravated astrocyte pyroptosis compared with the vehicle group. The secretion of pro-inflammatory cytokines, such as IL-1β and IL-18, was increased by rLCN2 induction (Fig. 8a, b). MCC950 is a selective and effective inhibitor of the NLRP3 inflammasome. An equivalent of 50 μg/kg MCC950 was intraperitoneally injected into mice immediately after the MCAO surgery. As expected, MCC950 significantly inhibited NLRP3 inflammasome activation (Fig. 8c, d, *P* < 0.001). The exacerbation of astrocyte pyroptosis induced by rLCN2 was remarkably reversed by MCC950. Taken together, these findings suggested that LCN2/24p3R mediates pyroptosis in astrocytes via the NLRP3 inflammasome after cerebral ischemia/reperfusion injury. Moreover, restraining astrocyte pyroptosis could minimize neurological impairment (Fig. 8e).

## Discussion

**Fig. 9:**
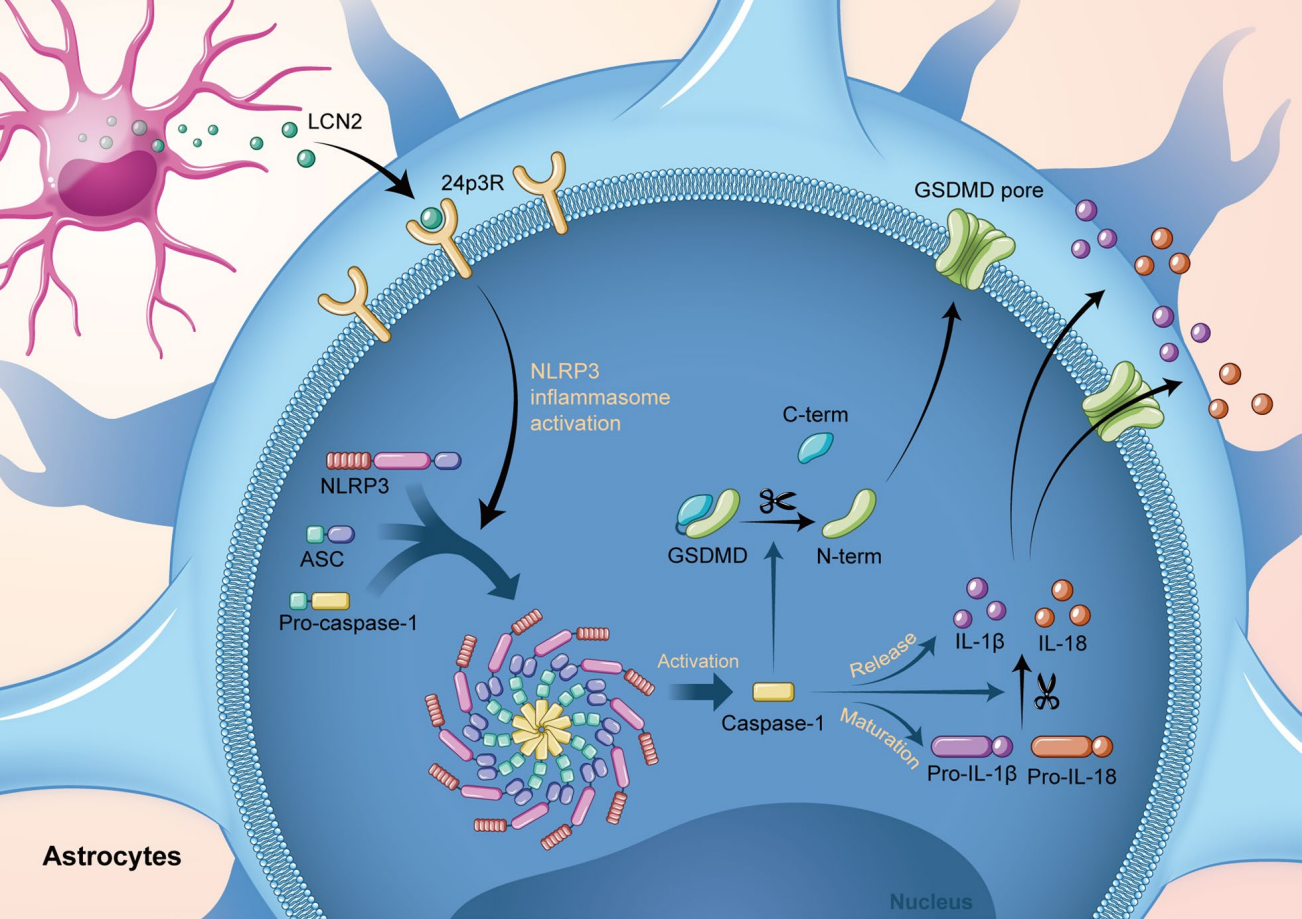
LCN2, secreted by astrocytes during brain ischemia/reperfusion injury, binds to 24p3R on the membrane of astrocytes. This process activates the NLRP3 inflammasome. Pro-caspase-1 was subsequently cleaved into caspase-1. GSDMD^Nterm^ cleaved by caspase-1 polymerized on the cell membrane, forming GSDMD^Nterm^ pores and causing programmed inflammatory cell death, pyroptosis. Activated caspase-1 promotes the maturation and release of pro-inflammatory cytokines, including IL-1β and IL-18

The present study identifies LCN2 as a critical regulator of astrocyte pyroptosis following cerebral ischemia/reperfusion injury. LCN2 levels were increased in astrocytes of the peri-infarction area after MCAO. Genetic knockout of *Lcn2* improved the short- and long-term outcomes of MCAO mice. Mechanistically, we verified the colocalization of LCN2 and 24p3R in astrocytes, providing a physical basis for their association. Pyroptosis in astrocytes was alleviated with an astrocyte-specific AAV-GFAP-24p3Ri, which could be reversed by a NLRP3 inflammasome activator. Similarly, re-expression of LCN2 in *Lcn2*^−/−^ mice aggravated GSDMD-mediated pyroptosis. This detrimental effect could be mitigated by 24p3R interference or NLRP3 inhibition. Collectively, in cerebral ischemia/reperfusion injury, LCN2 binds to 24p3R on the astrocyte membrane and facilitates NLRP3 activation, which ultimately triggers astrocyte pyroptosis and pro-inflammatory effects (Fig. 9). These findings provide new insights into the critical role of LCN2 in neuroinflammation after cerebral ischemia.

It is widely accepted that neuroinflammation is a vital secondary injury following stroke. The multiprotein complexes termed “inflammasomes” were first described by Martinon et al. [39], and are involved in the initiation of the inflammatory response [40]. Typically, inflammasomes consist of three components: a cytosolic pattern-recognition pattern (PRP)-sensing damage-associated molecular patterns (DAMPs) released from dead cells, the caspase-1 protease, and an adaptor protein integrating the two components [41]. Hitherto, the inflammasomes NLRC4, NLRP1, NLPR3, NLRP6 and AIM have been implicated in ischemic stroke [12, 13, 42]. Among these, the NLRP3 inflammasome is the most studied regulator of caspase-1 activation related to sterile inflammation in neurological diseases [43, 44]. In addition, the NLRP3 protein is upregulated following ischemic stroke. Inhibition of NLRP3 inflammasome activation improves neurological function [45, 46]. A report suggested that the NLRP3 transcript is not apparently expressed in astrocytes at baseline but could be induced by NLRP3-activation stimuli, including recombinant Aβ [47]. NLRP3 inflammasome activation initiates pro-inflammatory programmed cell death, termed pyroptosis [48]. Briefly, DAMPs released from damaged cells are recognized by pattern-recognition receptors (PRRs) to induce NLRP3 inflammasome activation, followed by pro-caspase-1 maturation. Gasdermins are executors of pyroptosis, represented by GSDMD [49]. Caspase-1 cleaves full-length GSDMD into GSDMD^Nterm^, whose polymerization forms the characteristic membrane pores of pyroptosis [9, 50–52]. However, factors triggering NLRP3 inflammasome activation in ischemic stroke remain unknown. Activated astrocytes in ischemic stroke could respond to inflammatory signals and promote inflammation. Accumulating evidence demonstrates that astrocyte pyroptosis is involved in the pathological process of cerebral ischemia/reperfusion. Suppressing NLRP3-mediated pyroptosis of astrocytes by hispidulin improves neurological symptoms and decreases the secretion of pro-inflammatory cytokines, infract volume and brain edema [11]. Pioglitazone conferred a neuroprotective function against astrocyte pyroptosis in brain injury [10]. Our results substantiated that the NLRP3 inflammasome was activated in astrocytes, causing astrocyte pyroptosis during ischemic stroke. When NLRP3 activation was inhibited, astrocyte pyroptosis was suppressed, suggesting that NLRP3 initiation is essential in astrocyte pyroptosis following cerebral ischemia/reperfusion injury.

LCN2 is an acute-phase protein secreted by activated astrocytes under diverse brain injury conditions [16]. LCN2 exerts an autocrine effect on astrocytes. To date, studies have focused on the role of LCN2 on astrocytes in reactive astrocytosis. Astrocytes have two distinct activation phenotypes, i.e., classic activation (pro-inflammatory) and alternative activation (anti-inflammatory). LCN2 auto-secreted from activated astrocytes in turn stimulates the classic activation and exerts an inflammatory effect [18, 53]. In a previous study, we revealed the requirement of LCN2 for the classic activation of astrocytes using *Lcn2*^−/−^ mice [19]. 24p3R is considered an LCN2 receptor. Devireddy et al. reported a specific cell-surface receptor of LCN2, 24p3R. Moreover, LCN2 is a secreted protein that needs to enter the cell through an endocytic mechanism, indicating that LCN2 has receptors on the cell surface. In another study, HeLa cell lines stably expressing 24p3R were derived, and ectopic expression of 24p3R endowed the cells with the ability to bind and internalize LCN2. These results revealed a role for LCN2/24p3R in mediating cell apoptosis [54]. Another work conducted biochemical studies based on nuclear magnetic resonance (NMR) to assess the interaction between LCN2 and its putative cell receptor 24p3R. The findings revealed that the N-terminal region of 24p3R is a soluble extracellular domain that is altered with a preferential interaction with LCN2 in its apo state in vitro [55]. LCN2/24p3R is widely expressed in multiple cells of the central nervous system and participates in various pathophysiological processes. Previous studies have demonstrated the colocalization of LCN2 and 24p3R using IF in neurons [56], astrocytes, microglia [57], oligodendrocytes [58], epithelial cells [59] and endothelial cells [20]. Current studies have identified an interaction between LCN2 and 24p3R.

We further expanded the function of LCN2/24p3R to cell pyroptosis and found that LCN2 triggers pyroptosis in astrocytes via binding to 24p3R; the generation of pro-inflammatory cytokines and neurological impairment were alleviated by *Lcn2* gene knockout. LCN2 can enhance inflammasomes through astrocytes/microglia activation and neutrophil infiltration [21]. Additionally, LCN2 may exert a direct neurotoxic effect by binding to the receptor on neurons [20]. The current study showed that LCN2-mediated astrocytic pyroptosis caused neuron damage. Herein, we discussed the possible mechanisms for further investigation. The astrocyte–neuron crosstalk contributes to the pathological process of various neurological diseases, and the neurotoxic effects of activated astrocytes have been widely studied [60–62]. Among these, the effect of astrocytic pyroptosis on neurons has attracted increasing attention. In a sepsis model, astrocyte pyroptosis increased the release of pro-inflammatory cytokines (IL-1β and IL-18), resulting in neuron damage [63]. Another study found that pyroptosis, especially in astrocytes, promotes the disruption of blood–brain barrier integrity and the accumulation of toxic Aβ, ultimately leading to neuronal death after I/R injury [64]. An in vitro study demonstrated that astrocyte pyroptosis decreases neuronal viability and aggravates neuronal apoptosis through caspase-1 activation [13]. Therefore, aggravated neuroinflammation and neuronal damage may increase infarct volume by MCAO.

Furthermore, we discussed the potential adaptor mediating LCN2/24p3R with downstream pyroptosis. In a nonalcoholic steatohepatitis (NASH) model, high circulatory LCN2 activated 24p3R in the brain and induced the release of high mobility group box 1 (HMGB1). This molecule is a toll-like receptor 4 (TLR4) ligand that induces oxidative stress by stimulating the NOX-2 signaling pathway (activated p65 protein of the NF-κΒ complex). Then, the HMGB1-derived downstream signaling pathway activates the NLRP3 inflammasome [65]. Another study validated the effect of LCN2 on cardiac dysfunction. After binding to its receptor 24p3R, LCN2 disrupts the association of Beclin-1 and HMGB1, and the released HMGB1 alters autophagic flux. On the other hand, HMGB1 induces an inflammatory response by binding to TLR4 receptors. LCN2 induces mitochondrial dysfunction and subsequent increase in ROS or renders mitochondrial DNA (mtDNA) conducive to priming and activation of the NLRP3 inflammasome [66]. LCN2 also plays a role in the pathophysiology of inflammatory bowel disease (IBD) and upregulates the NLRP3 inflammasome via NF‐κB activation [67]. A positive correlation was established between NLRP3 and the *Lcn2* gene in the adipose tissue. In summary, NLRP3 inflammasome activation is associated with TNFα-mediated adipocyte dysfunction. Lcn2 is the key mediator of the adipocyte–macrophage crosstalk, which serves as the second signal for NLRP3 activation [68]. In future studies, we aim to verify the mechanism underlying this phenomenon.

In summary, the current findings support a pro-pyroptotic role for LCN2 in cerebral ischemia/reperfusion injury. Inhibiting LCN2-induced astrocyte pyroptosis may be a promising therapeutic target for ischemia stroke management.

## Supplementary Information

**Additional file 1: Figure S1.** Astrocyte activation mainly occurs in penumbra after MCAO operation. **Figure S2.** After OGD, the secretion of LCN2 increases and then decreases, but no significant differences were detected in astrocyte cell viability. **Figure S3.** LCN2 and 24p3R are well colocalized in astrocytes. **Figure S4.** Astrocytic adverse effects on neurons are mediated by LCN2. **Figure S5.** Immunofluorescence negative control staining of GFAP. **Figure S6.** Pyroptosis also occurs in microglia and neurons post-MCAO. **Figure S7.** NLRP3 inflammasome is activated in microglia and neurons during cerebral ischemia/reperfusion injury.

## Data Availability

The data that support the findings of this study are available from the corresponding author upon reasonable request.

## Abbreviations

LCN2: Lipocalin-2
MCAO: Middle cerebral artery occlusion
O/R: Oxygen–glucose deprivation and reoxygenation
AAV-GFAP-24p3Ri: Astrocyte-specific adeno-associated virus
Nig: Nigericin sodium salt
rLCN2: Recombinant lipocalin-2
CCA: Common carotid artery
ECA: External carotid artery
ICA: Internal carotid artery
MCA: Middle cerebral artery
TTC: 2,3,5-Triphenyltetrazoliumchloride
PFA: Paraformaldehyde
mNSS: Modified Neurologic Severity Score
MWM: Morris water maze
AAV: Adeno-associated virus
HBSS: Hank’s balanced salt solution
DMEM: Dulbecco’s modified Eagle medium
PDL: Poly-d-lysine
PBS: Phosphate buffer saline
OGD: Oxygen–glucose deprivation
WB: Western blotting
SDS-PAGE: Sodium dodecyl sulfate-polyacrylamide gel electrophoresis
PVDF: Polyvinylidene difluoride
IF: Immunofluorescence
BSA: Bovine serum albumin
TUNEL: TdT-mediated dUTP nick-end labeling
qPCR: Real-time quantitative PCR
AAV-GFAP-24p3Ri: Astrocyte-specific adeno-associated virus-24p3Ri
PRP: Pattern-recognition pattern
DAMPs: Damage-associated molecular patterns
PRR: Pattern-recognition receptor
