# Supplementary material for: Lipocalin-2-mediated astrocyte pyroptosis promotes neuroinflammatory injury via NLRP3 inflammasome activation in cerebral ischemia/reperfusion injury
Source: J Neuroinflammation. 2023 Jun 23;20:148. doi: 10.1186/s12974-023-02819-5 (PMC10288712; doi:10.1186/s12974-023-02819-5)
Supplement: Supplementary file 1 — Additional file 1: Figure S1. Astrocyte activation mainly occurs in penumbra after MCAO operation. Figure S2. After OGD, the secretion of LCN2 increases and then decreases, but no significant differences were detected in astrocyte cell viability. Figure S3. LCN2 and 24p3R are well colocalized in astrocytes. Figure S4. Astrocytic adverse effects on neurons are mediated by LCN2. Figure S5. Immunofluorescence negative control staining of GFAP. Figure S6. Pyroptosis also occurs in microglia and neurons post-MCAO. Figure S7. NLRP3 inflammasome is activated in microglia and neurons during cerebral ischemia/reperfusion injury. [file 12974_2023_2819_MOESM1_ESM.docx]

**Additional file 1**

**Figure S1. Astrocyte activation mainly occurs in penumbra after MCAO operation. (a)** Astrocytes activation after MCAO surgery was detected by immunofluorescence staining of GFAP (green). Scale bar = 1000 μm in the whole brain scan and 20 μm in the enlarged view. **(b, c)** Levels of GFAP expression in penumbra were assessed by Western blotting at 24 h and 72 h after MCAO. Data are shown as mean ± SD.; n = 3 per group; ^***^*P* < 0.001, ^*^*P* < 0.05 *vs*. sham-operated group; ^###^*P* < 0.001.


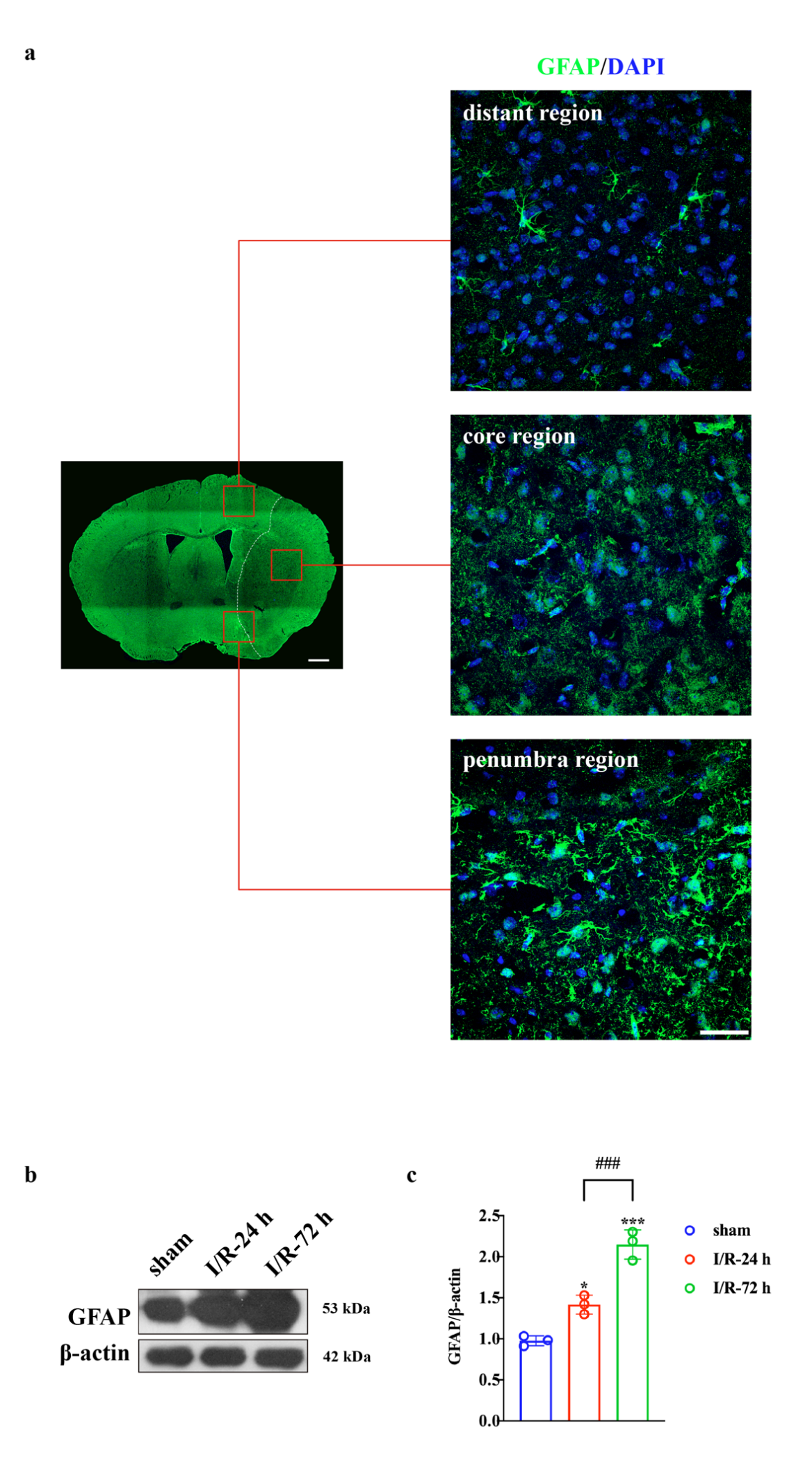


**Figure S2. After OGD, the secretion of LCN2 increases and then decreases, but no significant differences were detected in astrocyte cell viability. (a)** ELISA detection of LCN2 secretion in the supernatants. **(b)** Cell viability was assessed by LDH assay. All data are shown as mean ± SD.; n = 5 per group; ^***^*P* < 0.001 *vs*. control group; ^###^*P* < 0.001, ^$$^*P* < 0.01.


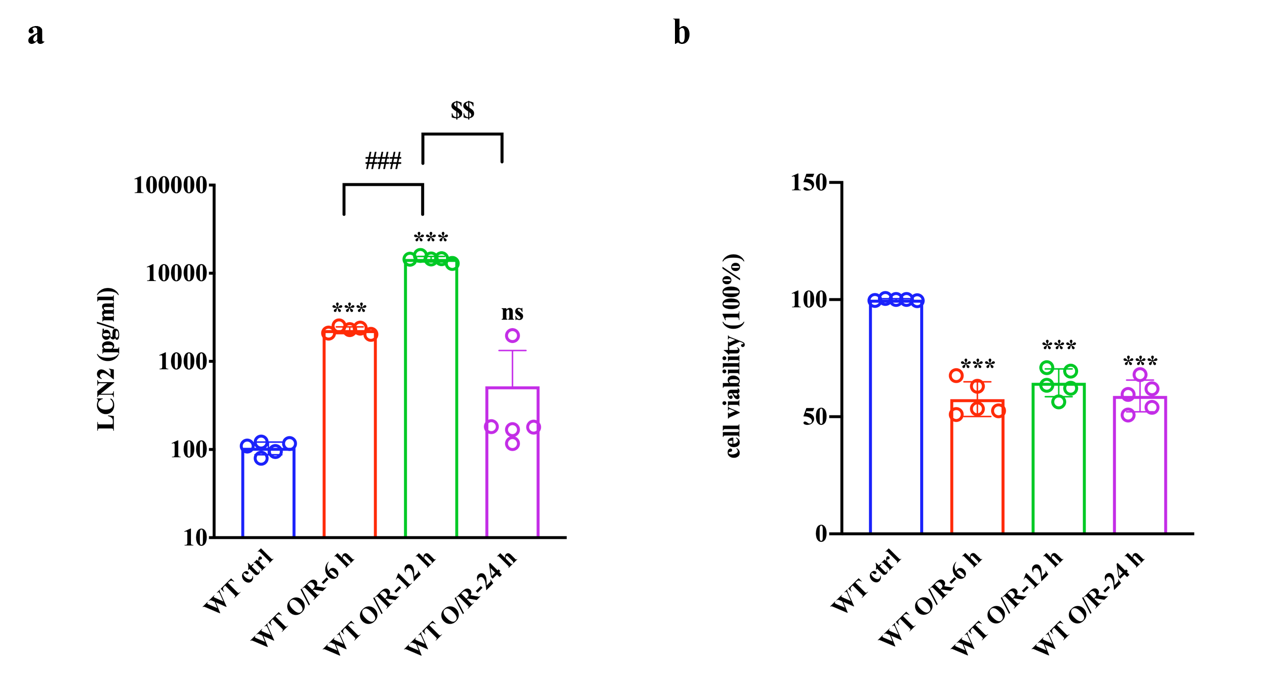


**Figure S3. LCN2 and 24p3R are well colocalized in astrocytes.** Triple immunostaining of GFAP, LCN2, and 24p3R. Scale bar = 50 μm.


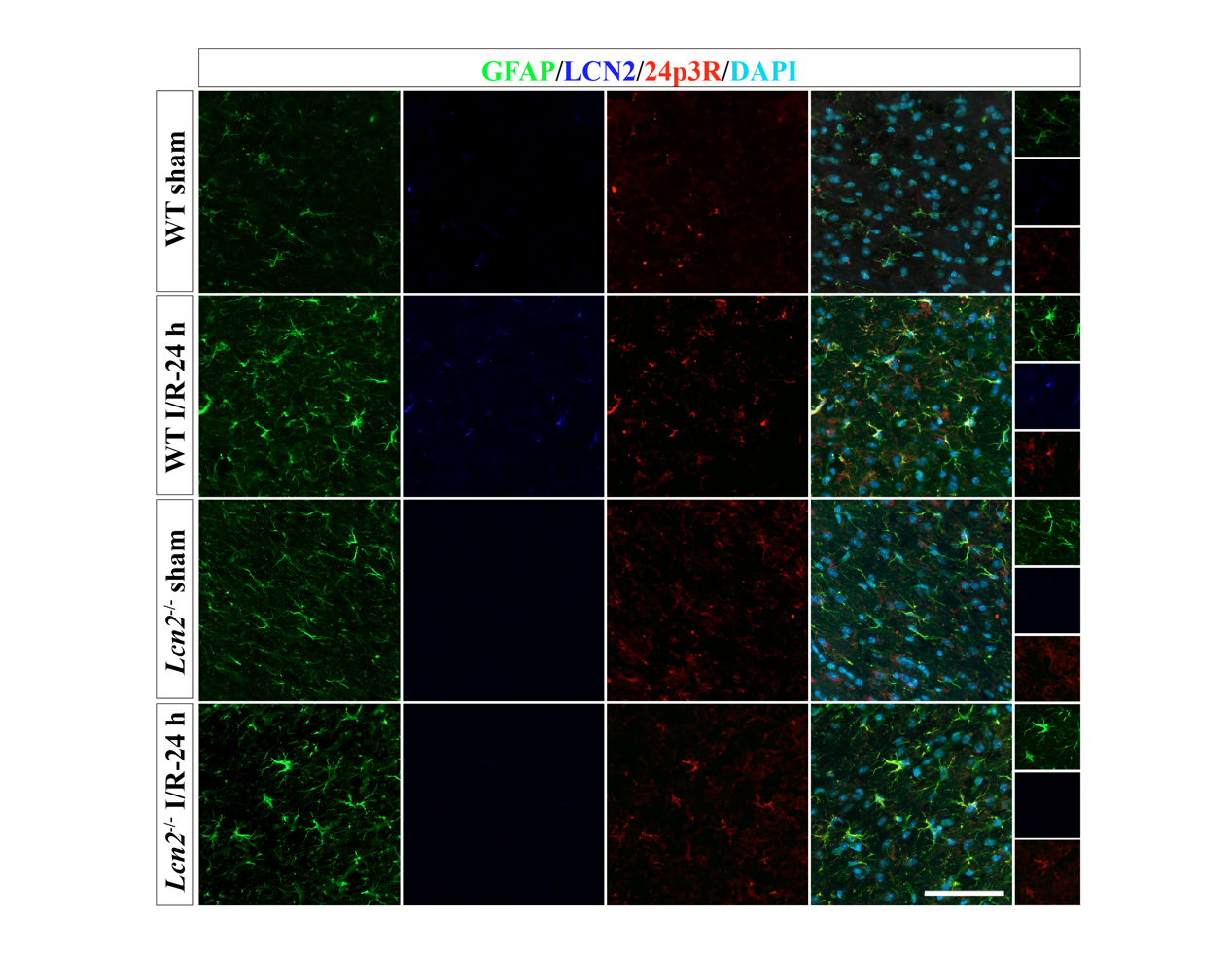


**Figure S4. Astrocytic adverse effects on neurons are mediated by LCN2. (a)** A co-culture model of neurons and astrocytes. **(b, c)** Double staining of NeuN (green) and TUNEL (red). Scale bar = 50. ^***^*P* < 0.001 *vs*. control group; ^###^*P* < 0.001, ^$$$^*P* < 0.001.


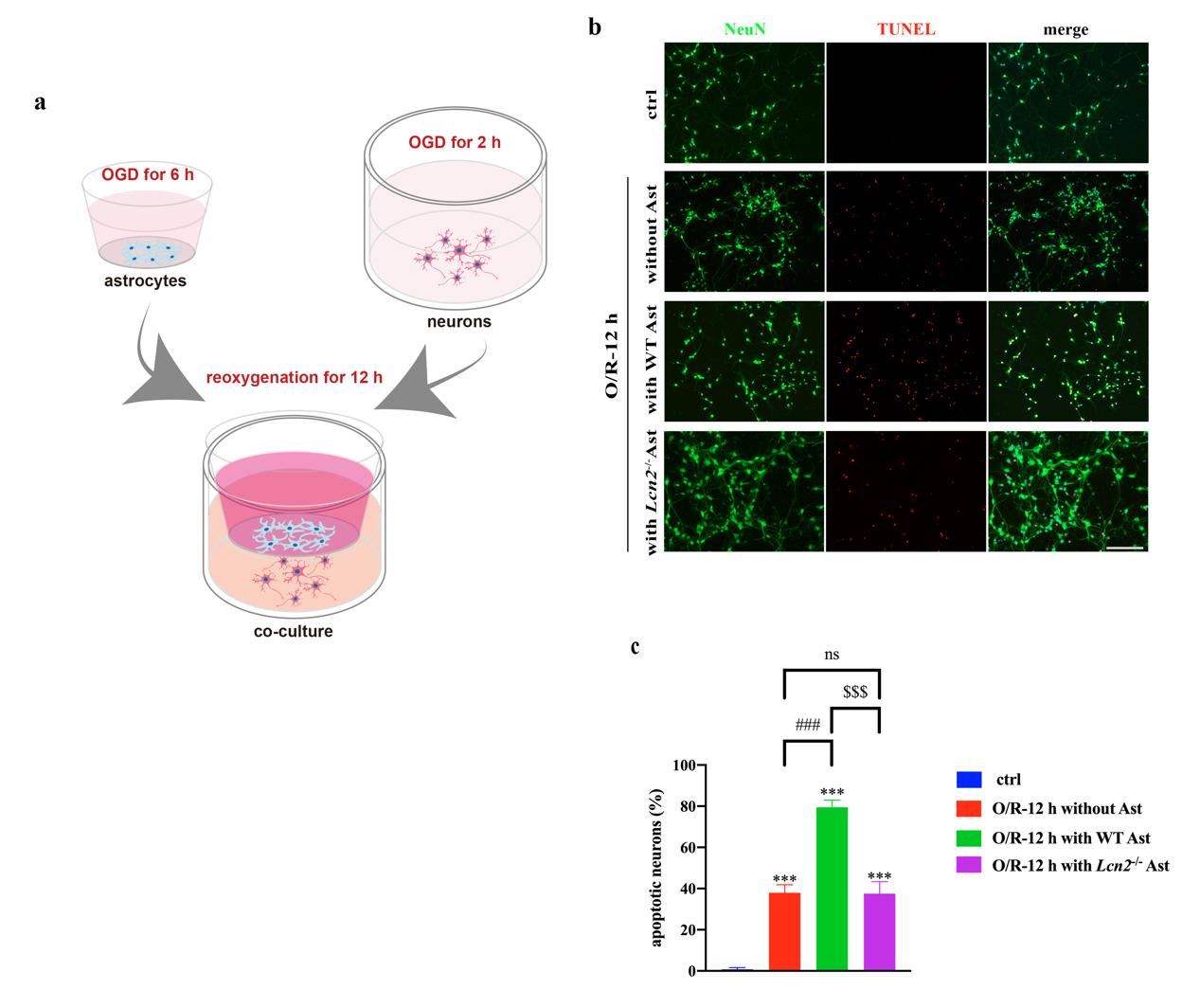


**Figure S5. Immunofluorescence negative control staining of GFAP.** Brain slices were stained with secondary antibody (Alexa Fluor 488) only. Scale bar = 50μm.


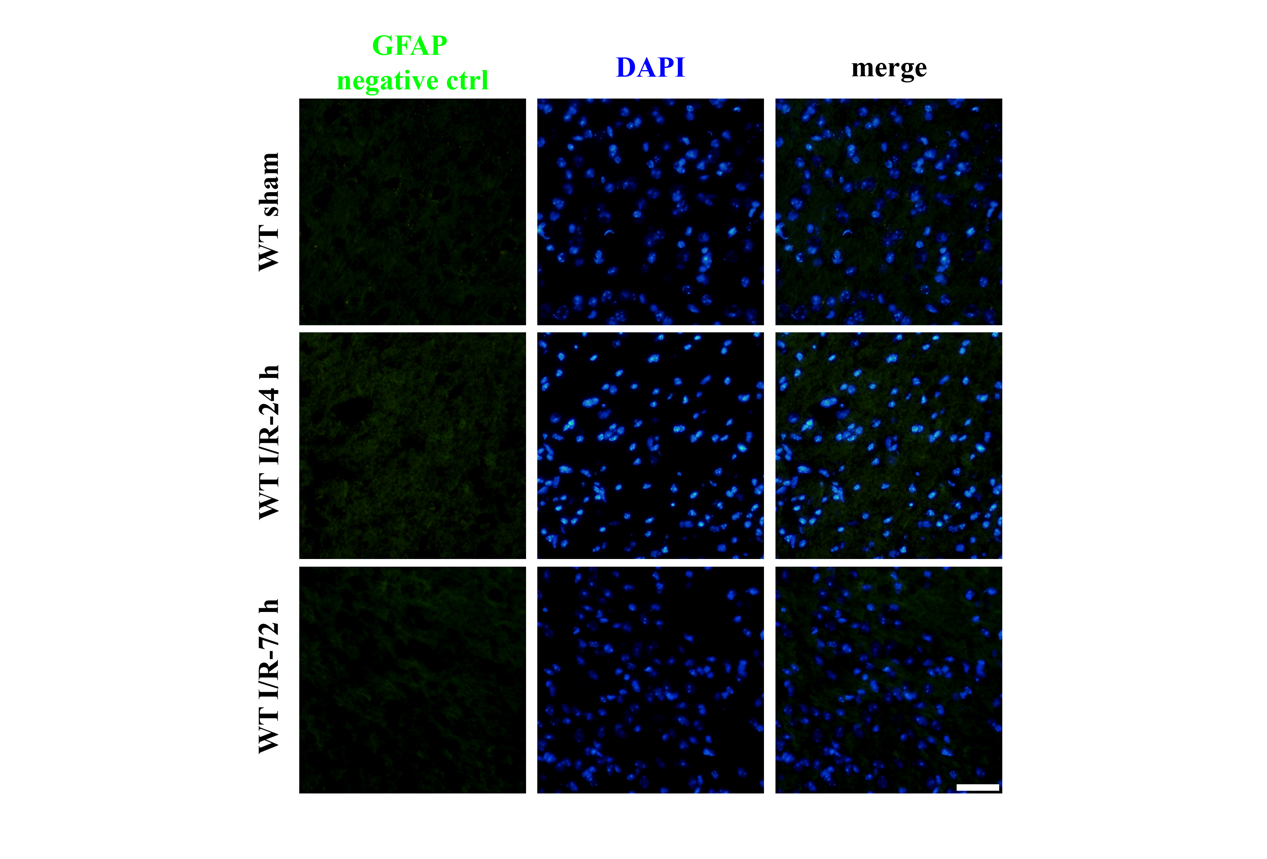


**Figure S6. Pyroptosis also occurs in microglia and neurons post MCAO. (a)** Immunofluorescence double staining of Iba-1 (green) and GSDMD (red); **(b)** Immunofluorescence double staining of NeuN (green) and GSDMD (red). Scale bar = 50 μm.

**
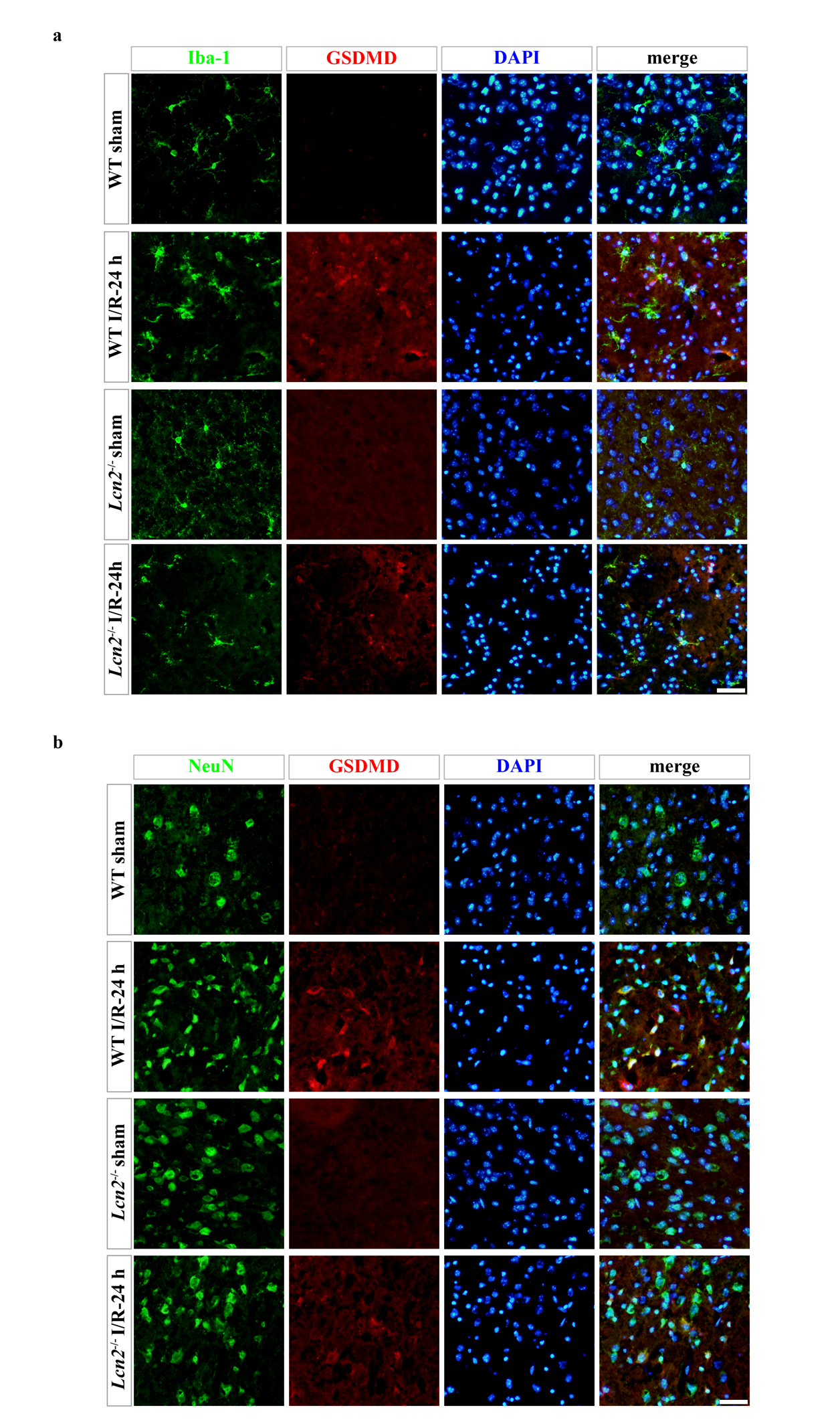
**

**Figure S7. NLRP3 inflammasome is activated in microglia and neurons during cerebral ischemia/reperfusion injury. (a)** Immunofluorescence double staining of Iba-1 (green) and NLRP3 (red), ASC(red), caspase-1 (red); **(b)** Immunofluorescence double staining of NeuN (green) and NLRP3 (red), ASC(red), caspase-1 (red). Scale bar = 50 μm.

**
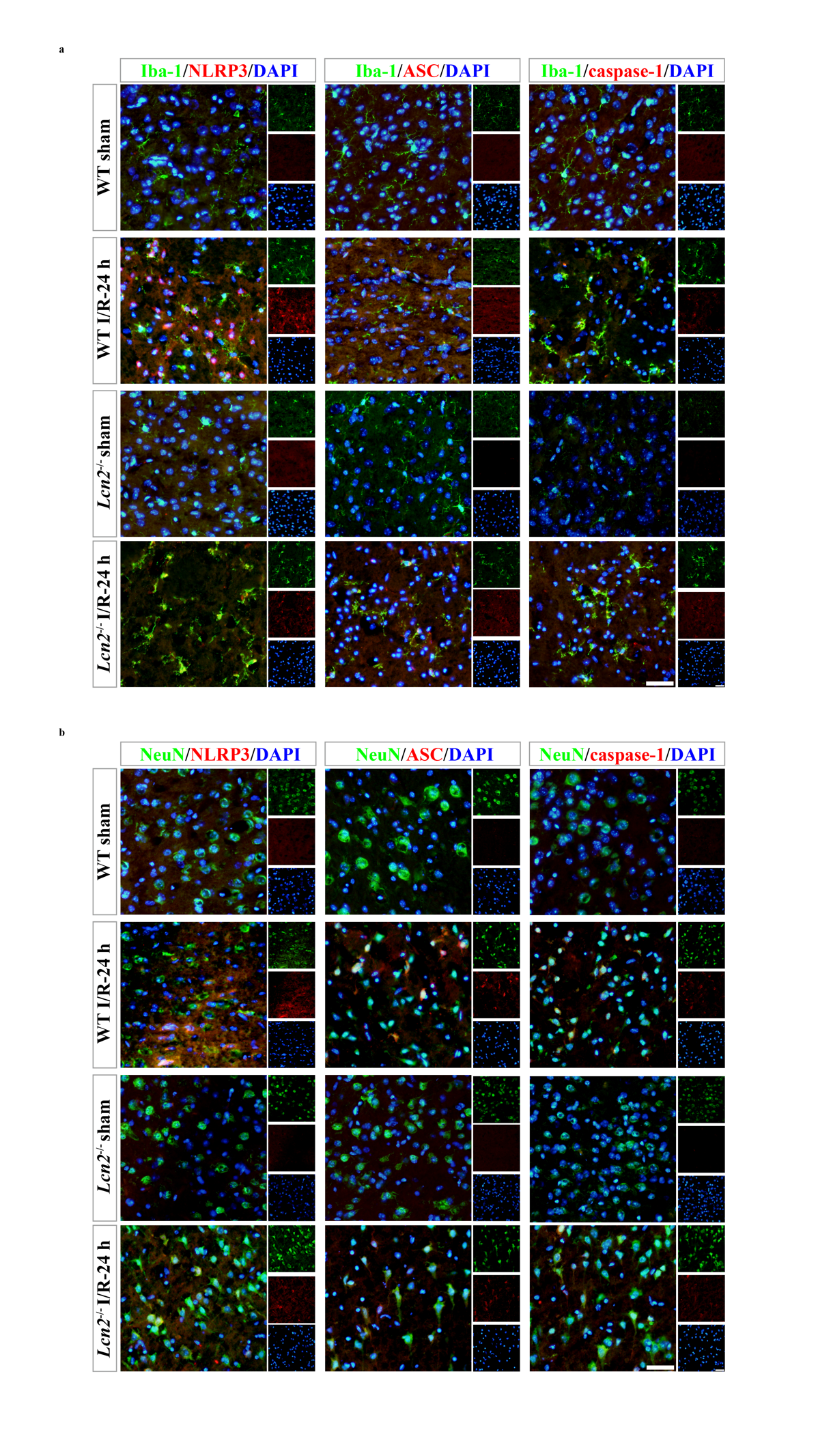
**
